# Comprehensive physicochemical, biophysical, and *in vitro* characterization of lung surfactant SP-A peptidomimetics

**DOI:** 10.1039/d4pm00265b

**Published:** 2025-05-06

**Authors:** David Encinas-Basurto, Priya Muralidharan, M. D. Saiful Islam, Ernest L. Vallorz, Stephen M. Black, Monica Kraft, Julie G. Ledford, Heidi M. Mansour

**Affiliations:** a The University of Arizona College of Pharmacy Tucson AZ USA; b University of Sonora Mexico; c The University of Arizona College of Medicine Tucson AZ USA hmansour@fiu.edu jledford@arizona.edu; d Florida International University, Center for Translational Science Port St. Lucie FL USA; e The University of Arizona Health Sciences Asthma and Airway Disease Research Center Tucson AZ USA; f The University of Arizona, the BIO5 Research Institute Tucson AZ USA; g Department of Medicine, Icahn School of Medicine at Mount Sinai, Mount Sinai Health System New York NY USA

## Abstract

Surfactant protein-A (SP-A) is an endogenous and essential lung surfactant-specific protein that is integral to pulmonary immunity, including inhibition of asthma exacerbations. This study aims to comprehensively characterize two peptides (10-AA and 20-AA) of SP-A which confer activity similar to the full-length oligomeric SP-A protein. Spectroscopic and chromatographic analyses revealed that the phosphate (PS) and acetate (AC) salts exhibited distinct solubility and log *P* partitioning behavior, impacting their physicochemical properties. MD simulations and circular dichroism showed that SP-A 10-AA initially adopts an α-helical structure but loses helicity over time, while SP-A 20-AA remains disordered. Differential scanning calorimetry confirmed variations in thermal stability between salt forms and zeta potential measurements showed that PS salts had a more negative surface charge, potentially influencing membrane interactions. *In vitro* studies showed high cell viability (>90%) and stable TEER values at the air–liquid interface, confirming biocompatibility and potential epithelial permeability. These findings provide crucial insights into the structural and functional properties of SP-A peptides, supporting their potential as therapeutic agents for pulmonary diseases.

## Abstract 2

This study characterizes the physicochemical properties of SP-A-derived peptides in acetate and phosphate forms, evaluating their impact on lung cell viability. Findings provide insights into their stability and potential as inhalable therapeutics.

## Graphical abstract

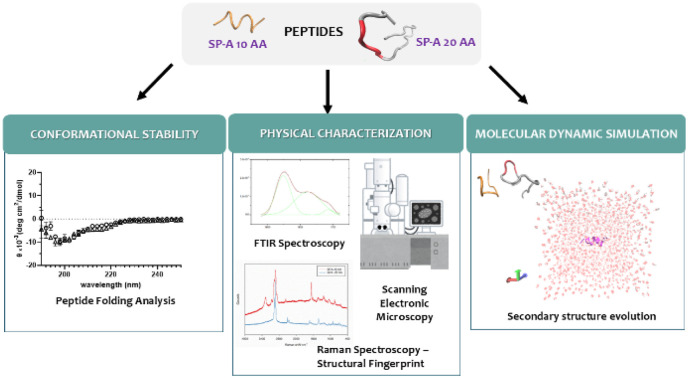

## Introduction

Surfactant Protein A (SP-A), one of the major protein constituents of pulmonary surfactant, is an important mediator of pulmonary host defense. SP-A is encoded by two genes, *SP-A1* and *SP-A2*, and forms a highly oligomeric octodecamer structure. Specific to asthma, it was previously shown that SP-A function is defective, which could arise from genetic variation.^1–3^ Hence, a 10-amino acid (AA) active region of SP-A2 has been identified that significantly reduces phenotypes associated with asthma in human cells and in murine models.^4^ This 10-AA (1.25 kDa) size SP-A peptide is an ideal candidate to package into an inhaler for airway delivery.

Inhaled medicine has become the preferred route of administration for targeted drug delivery directly to the lung tissue by a noninvasive route for local and systemic action.^5^ Inhaled medicines as marketed products are the first-line therapies used clinically in the treatment and preventative management of several pulmonary diseases of global health importance (*i.e.* asthma, COPD, cystic fibrosis, pulmonary fibrosis, and pulmonary infections) and noninvasive systemic therapy through the pulmonary route (*i.e.* Afrezza® inhaled insulin dry powder inhaler and Adasuve® inhaled antipsychotic marketed pharmaceutical products).

Peptides and proteins are complex macromolecules. Consequently, the first step in development of a protein or peptide-based drug product involves comprehensive characterization to understand their chemical structure and physicochemical properties that pertains to its biological activity and stability. The advancement in analytical chemistry field provides a plethora of techniques for evaluation of these biopharmaceuticals in the different stages of product development. Infrared spectroscopy, Raman spectroscopy, circular dichroism, solid state nuclear magnetic resonance are powerful techniques for structure elucidation. Thermal analytical techniques like differential scanning calorimetry, thermogravimetry and X-ray diffraction is used to probe thermal behavior, molecular mobility, kinetics of solid state stability as well as extent of crystallinity of peptide.^6,7^ It is imperative to identify these properties of peptide since it can affect the processability, chemical stability and eventually *in vivo* efficacy of these biomolecules. Therefore, a combination of these techniques is utilized to thoroughly characterize the peptide before and after development of the final formulation or drug product.

The work presented in this manuscript is the first report to the Authors’ knowledge on the comprehensive physical and biophysical characterization, *in vitro cellular* properties, *in silico* molecular modeling, and *in vitro* human cell properties of the innovative SP-A 10-AA and SP-A 20-AA peptides as two different salt forms (phosphate (PS) and acetate (AC)). The results presented in this study will serve as a reference for the physicochemical properties of the two SP-A peptides, which can be compared with the final drug formulation intended for lung inhalation.

## Materials and methods

### Materials

SP-A 10-AA has an amino acid sequence of Lys-Glu-Gln-Cys-Val-Glu-Met-Tyr-Thr-Asp (KEQCVEMYTD) and SP-A 20-AA sequence is Pro-Ala-Gly-Arg-Gly-Lys-Glu-Gln-Cys-Val-Glu-Met-Tyr-Thr-Asp-Gly-Gln-Trp-Asn-Asp (PAGRGKEQCVEMYTDGQWND). The PS and AC 10 AA, PS and AC 20 AA peptides with molecular mass of 1245.38 g mol^−1^ and 2284.45 g mol^−1^ respectively, were synthesized by GenScript (Piscataway, NJ) with >98% purity. Hydranal®-Coulomat AD, trifluoroacetic acid (TFA) and 1-Octanol were obtained from Sigma-Aldrich (St Louis, MO). Phosphate buffered saline (PBS) pH 7.4 (1×) was obtained from Life Technologies (Carlsbad, CA) and Emprove (absolute ethanol) USP grade was obtained from VWR (Radnor, PA). Methanol and Acetonitrile (ACN) HPLC grade were obtained from Thermo Fisher Scientific (Waltham, MA). Resazurin sodium salt was from Thermo Fisher Scientific (Waltham, MA). The peptides were stored in sealed glass desiccators over indicating Drierite/Drierite™ desiccant at −20 °C under ambient pressure. Other chemicals were stored under room conditions.

### Methods

#### Comprehensive morphological and elemental characterization using scanning electron microscopy and energy dispersive X-ray spectroscopy (SEM/EDX)

The peptide surface was imaged using SEM FEI Inspect S (Brno, Czeck republic) scanning electron microscope similar to the condition that was previously reported.^8,9^ Briefly, the peptide powders were mounted on a double coated carbon conductive tabs on aluminum stub, which was gold coated for 90 seconds under argon plasma using Anatech Hummer 6.2 (Union city, CA, USA). Images were captured using 30 kV voltage at various magnifications. EDX was performed using ThermoNoran systems Six (Thermo Scientific, Waltham, MA, USA) by adjusting spot size until a dead time of 20–30 was obtained.

#### Circular dichroism spectroscopy for secondary structure analysis of SP-A peptides

Structural properties of SP-A PS and AC salt peptides (10-AA and 20-AA) were studied using Olis™ DSM 20 CD Spectrophotometer (Olis, Inc., Bogart, CA). The experimental conditions were optimized following previously reported methods.^10,11^ Peptides were prepared in DI-water at 380 and 240 μM for 10-AA and 20-AA peptides respectively. Far-ultraviolet CD spectra of peptides were obtained using 0.5 mm pathlength cylindrical cells at two temperatures of 25 °C and 37 °C. Each scan was recorded with an average of three replicate scans from 250 to 190 nm at 2 nm intervals, with an integration time of 10 s and a water baseline spectrum subtracted from each sample spectrum. Molar ellipticity was calculated using the mean residue molecular weight for each peptide as previously described.^10^

#### Differential scanning calorimetry for thermal stability and phase transition analysis

Thermal analysis of the SP-A peptides were performed using similar conditions to those previously reported.^8,9^ Briefly, a 1–10 mg mass of peptide powder was carefully weighed into T-Zero® DSC hermetic pans (TA Instruments, New Castle, DE) that were hermetically sealed and scanned from 25.00 °C to 250.00 °C at a heating rate of 5.00 °C min^−1^ using a TA Q1000 Differential Scanning Calorimeter (DSC) (TA Instruments, New Castle, DE) with RSC90 automated cooling system. Nitrogen was used as the purging gas at a rate of 50 mL min^−1^.

#### X-ray powder diffraction for crystallinity and structural analysis

The XRPD analysis of the peptides were conducted according to the conditions reported in previous studies.^8,9^ Briefly, the peptide powders were scanned from 5–70° 2*θ* at a scan rate of 2.00° min^−1^ at room temperature with a Cu Kα radiation (45 kV, 40 mA, and *λ* = 1.5406 Å) from PanAnalytical X'pert diffractometer (PANalytical Inc., Westborough, MA).

#### Attenuated total reflectance–fourier transform infrared spectroscopy (ATR-FTIR) for structural and chemical analysis

Vibrational spectroscopy was conducted for molecular fingerprinting of the peptides, using similar conditions previously reported.^8,9^ Briefly, the peptides in their solid state was scanned from 400 cm^−1^ to 4000 cm^−1^ with an average of 64 scans using Nicolet Avatar 360 FTIR spectrometer (Varian Inc., CA) equipped with a DTGS detector and a Harrick MNP-Pro (Pleasantville, NY).

#### Raman spectroscopy for molecular fingerprinting and structural characterization

Raman spectroscopy was performed to acquire the molecular fingerprint for each peptide using similar conditions reported earlier.^8,9^ Briefly, Raman spectra were obtained at 512 nm laser excitation using Renishaw InVia Reflex (Gloucestershire, UK) at the surface using a 20× magnification objective on a Leica DM2700 optical microscope (Wetzlar, Germany) and equipped with a Renishaw inVia Raman system (Gloucestershire, UK). The laser power was varied to balance the signal-to-noise (S/N) ratio.

#### Zeta potential measurement for peptides charge

All samples were made using ultrapure water for dilution of buffers. Phosphate buffered saline (1×) and Normal Saline (0.85% w/v) were purchased from Gibco by Life Technologies and Thermofisher, respectively. Measurements were made by a Malvern Instruments Nano ZS, model ZEN3600 Zetasizer (Malvern, UK), using DTS1070 disposable cuvettes and the Smoluchowski model approximation of the Henry function. Peptide suspensions were prepared at 0.1 mg mL^−1^ and loaded into cuvettes by the diffusion barrier method.^12^ Samples were evaluated in triplicate after 30 minutes of equilibration at 25 °C.

#### Coulometric Karl Fischer titration for precise moisture content analysis

The conditions used to measure the residual water content was similar to previous reports.^8,9^ About 5–20 mg of the peptide was added to the titration vessel, TitroLine 7500 trace titrator (SI Analytics, Germany), containing Hydranal® Coulomat AD reagent and the titration was performed to measure the amount of water content in the powder.

#### *In vitro* solubility assessment for peptide formulation and bioavailability

*In vitro* solubility measurements were conducted using conditions earlier reported.^13^ Excess of peptide powder was added to known volume of pure solvent in a glass vial. The vial was allowed to rotate at ambient room temperature for 24 hours.^13^ The test solution was filtered using 0.2 μm PTFE or PVDF membrane filters for organic and aqueous solvents respectively. The filtered solution was chemically analyzed by HPLC using conditions described in the HPLC section below.

#### Octanol/water partition coefficient for lipophilicity and drug permeability assessment

Using similar conditions reported earlier,^14^ octanol/water partition coefficient (*P*) measurements were conducted. A mass of 3 mg of peptide powder was added to 3 mL of 1-octanol (1.5 mL) and water (1.5 mL) to create a 50 : 50 v/v octanol : water ratio in a glass vial and allowed to shake in temperature-controlled water bath at 37 °C. After 24 hours, the shaker was stopped and the vial was undisturbed for 24 hours to allow the solvents to separate. An aliquot (20 μL) of water phase and octanol phase was injected into HPLC to quantify the peptide concentration in each phase.

#### Theoretical isoelectric point (pI) and net charge calculation for peptide charge profiling

The pI and net charge of the SP-A 10-AA and SP-A 20-AA peptides were calculated using the ExPASy ProtParam tool.^15^ The amino acid sequences of each peptide were submitted to the ProtParam server (https://web.expasy.org/protparam/), which estimates the pI based on the Henderson–Hasselbalch equation and known p*K*_a_ values of ionizable groups in the peptide sequence. The net charge at pH 7.0 was also determined using the same tool, which considers the dissociation of acidic and basic residues at this pH.

#### High performance liquid chromatography (HPLC)

High-performance liquid chromatography was used to quantify the peptides. The analysis was performed with a Shimadzu LC-2010AHT HPLC system (Kyoto, Japan), with autosampler fitted to a 20 μL sampling loop and dual wavelength UV-Vis detector and reverse phase C_18_ Phenomenex Prodigy™column (Torrance, CA) with 250 × 4.6 mm, 5 μm particle size. The mobile phase consisted of (A) 0.065% TFA in water and (B) 0.05% TFA in ACN at a flow rate of 1.0 mL min^−1^. Analysis was performed using the following gradient elution method: 100% A to 100% B in 15 minutes, maintained at 100% B until 17.5 minutes. The percentage of A reached 100% in 19.5 minutes and maintained until 25 minutes. The column was maintained at 25 °C. The injection volume was 20 μL. The UV detector was set at 220 nm wavelength. The solvents used to dissolve 10-AA and 20-AA peptide analysis were PBS and pure water, respectively.

#### *In vitro* cell viability assessment for biocompatibility and cytotoxicity evaluation

The cell viability of three different human pulmonary cell lines from different lung areas representing the conducting airways, the mid-lung and deep lung respiratory regions of the small airways. They are A549 (human alveolar epithelial lung cells), H358 (human bronchioalveolar epithelial cells) and NCI-H441 (human papillary lung adenocarcinoma cells), were studied using the Resazurin assay. Pulmonary cell lines A549 (ATCC® CCL-185™), H358 (ATCC® CRL-5807™) and NCI-H441 (ATCC® HTB-174™) were purchased from the American Type Culture Collection ATCC® (Manassas, VA). As previously reported,^16^ A549 and H358 were grown in Dulbecco's modified eagle's medium (DMEM) Advanced 1× supplemented with 10% v/v fetal bovine serum (FBS), Pen-Strep (100 U mL^−1^, 100 μg mL^−1^), Fungizone® (0.5 μg mL^−1^ amphotericin B, 0.41 μg mL^−1^ sodium deoxycholate) and 2 mM l-glutamine obtained from Gibco® by Life Technologies (Thermo Fisher Scientific Inc., Waltham, MA). H441 cell line was grown in RPMI 1640 with l-glutamine Gibco® by Life Technologies (Thermo Fisher Scientific Inc., Waltham, MA).

The assay was conducted using similar previously reported conditions.^16^ Briefly, in Nunc™ MicroWell™ 96-well optical-bottom plates with polymer base (ThermoScientific Waltham, MA) each cell line was seeded at a concentration of 5000 cells in 100 μL per well (six wells, *n* = 6) and allowed to attach for 48 hours. The cells were treated with 100 μL volume of different concentrations (0.1–1000 μM) of the peptides dissolved in the respective media. After 72 hours, the plate was treated with 20 μL of 10 μM resazurin solution. After 3–4 hours, the fluorescence intensity was read at 514 nm (excitation) and 590 nm (emission) wavelengths using the Synergy H1 Multi-Mode Reader (BioTek Instruments, Inc., Winooski, Vermont). The corresponding growth media was used as control for each cell line. Relative viability was calculated as a ratio of fluorescence intensity of the sample to the control.
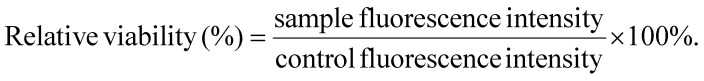


#### *In vitro* transepithelial electrical resistance (TEER) measurement for barrier integrity and permeability assessment

TEER was measured using H441 cell line in air–liquid interface (ALI) condition similar to the methods reported.^16,17^ Briefly, H441 cells were grown in T-75 culture flasks in an atmosphere of 5% CO_2_ at 37 °C. The cells were maintained in proliferation medium (RPMI 1640-Gibco) containing 10% fetal bovine serum (FBS) and 1% pen-Strep and 1% GlutaMAX. Once they were confluent (90%), cells were seeded onto 12-well Transwell inserts (Costar 3460, Corning, NY) at a density of 250 000 cells per well in proliferation medium (0.5 ml in the apical and 1.5 ml in the basolateral chambers). The seeding day was defined as Day 0 and the cells were allowed to attach. The basal media was changed every other day until the formation of a monolayer. After the cells reached confluence, the polarization medium was removed from the apical compartment, leaving the apical surface of the cells exposed to air *i.e.* air–liquid (ALI) culture condition. The polarization medium was made up of base medium RPMI 1640 containing 4% FBS, 1% penicillin–streptomycin, 1% GlutaMAX, 1% insulin-transferrin-selenium (ITS, Thermo Fisher, Auckland, New Zealand), and 200 nM dexamethasone (Sigma, Auckland, New Zealand). Medium was changed every two days. The maximum ALI TEER value expected was around 250 Ω cm^2^. Once they reached that value, cells were dosed with 100 μM of each peptide. TEER values were read before the treatment and 3 hours after treatment and every day to monitor the behavior of the monolayer.

#### *In silico* peptide modeling

Models were developed *in silico* using Molecular Modelling Pro Plus® version 13 software and ChemSite version 5.10 software (ChemSW, Accelrys Inc., San Diego, CA USA). Energy minimization was performed after each model were constructed by sequential addition of amino acids residues using Chemsite at α-helix and β-sheet conformation. Interatomic distances were measured after multiple iterations were made until no change in energy gradient were observed using Molecular Modelling Pro Plus® software applying MOPAC application.

#### Molecular dynamics simulation for structural stability and conformational analysis of SP-A peptides

The molecular dynamics (MD) simulations were performed to evaluate the structural stability and conformational behavior of the SP-A 10-AA (KEQCVEMYTD) and SP-A 20-AA (PAGRGKEQCVEMYTDGQWND) peptides. Initial three-dimensional structures were generated using I-TASSER website, selecting the highest-ranked models based on C-score and confidence level. The systems were prepared using CHARMM-GUI, where each peptide was solvated in a cubic box with explicit TIP3P water molecules, neutralized with 0.15 M NaCl, and parameterized using the CHARMM36m force field. Energy minimization was performed *via* the steepest descent algorithm, followed by 200 ps of equilibration under NVT and NPT ensembles at 310 K with the V-rescale thermostat and Parrinello–Rahman barostat. Production simulations of 10 ns were executed in GROMACS 2022 with periodic boundary conditions, a 2 fs timestep, and DSSP secondary structure analysis to monitor α-helix and β-sheet evolution were performed. These simulations provided insights into the peptides’ dynamic behavior.

#### Statistical analysis

All experiments were performed in minimum triplicate (*n* ≥ 3) unless otherwise mentioned. Numerical results are expressed as mean ± standard deviation obtained using Microsoft Excel 2016 (Redmond, WA). For statistical analysis, a one-way analysis of variance (ANOVA) was conducted to determine whether there were significant differences between experimental groups. When statistical significance was detected (*p* < 0.05), a Tukey's *post-hoc* test was performed to identify specific pairwise differences. Statistical significance is indicated in the figures using letters, where different letters denote statistically significant differences between treatments at the same concentration. All statistical analyses were performed using SPSS Statistics 27.

## Results

### Scanning electron microscopy/energy dispersive X-ray spectroscopy (SEM/EDX)

**Fig. 1:**
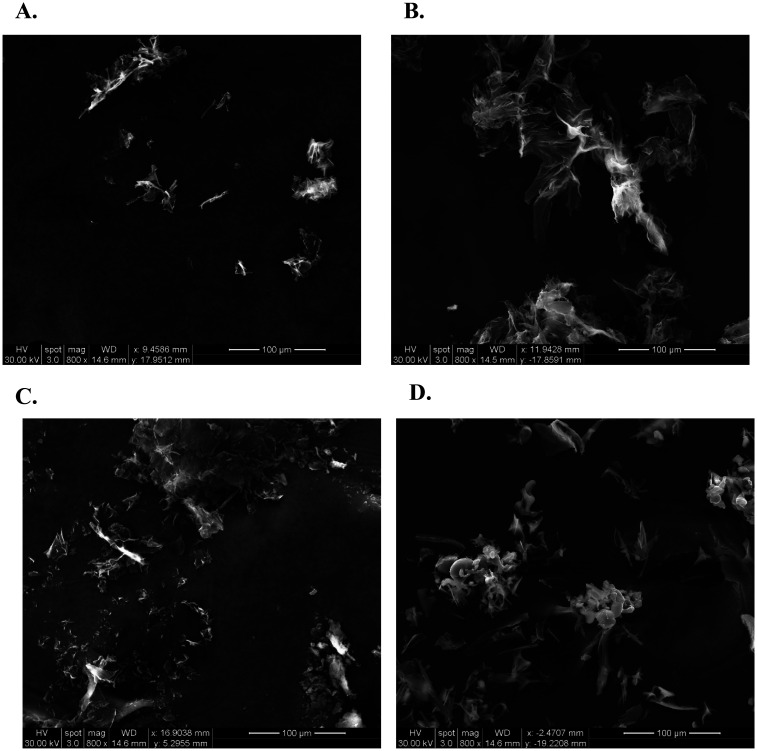
Scanning electron microscopy (SEM) images of SP-A peptides highlighting morphology variations between acetate (AC) and phosphate (PS) salts. (A) SP-A_10-AA PS shows dispersed aggregates, while (B) SP-A_10-AA AC forms fibrillar structures. (C) SP-A_20-AA PS presents loosely packed sheets, whereas (D) SP-A_20-AA AC forms compact clusters. Scale bars: 100 μm.

SEM is a tool to study the microstructural properties of biomolecules. It is important to study the microstructure of the peptides to understand the influence of different conditions that will be encountered in drug product development. The surface structure and morphology of the peptide/s could change with varying techniques of treatment. The microscopic image of both counter-ion SP-A peptides 10-AA and 20-AA can be observed in Fig. 1. SP-A peptides show a network which is representative of the complex folding of the peptides.

**Fig. 2:**
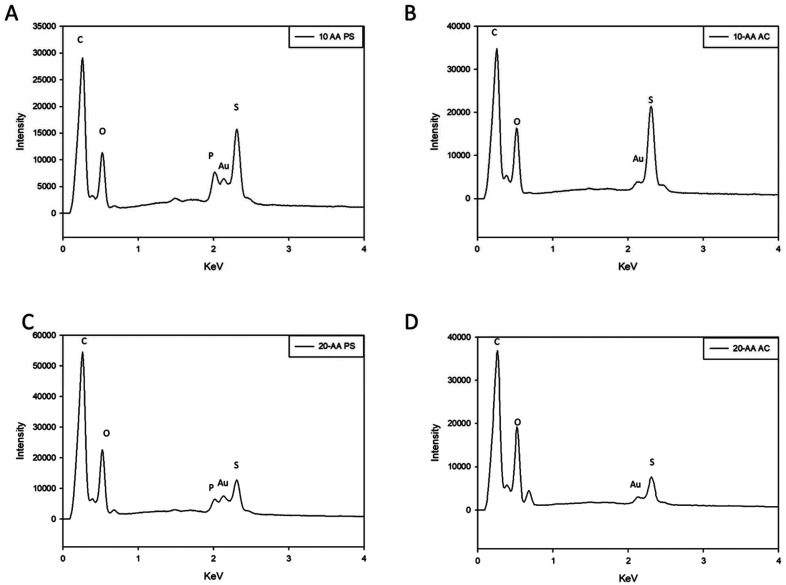
Energy dispersive X-ray (EDX) spectra of SP-A peptides: (A) SP-A 10-AA PS; (B) SP-A 10-AA AC; (C) SP-A 20-AA PS; and (D) SP-A 20-AA AC.

EDX is used to determine the molecular composition of the peptide that provides useful information about the atomic percent and stoichiometric ratios. The EDX analysis of the peptides showed peaks corresponding to carbon, oxygen, nitrogen, and sulfur for both peptides as seen from Fig. 2. Additionally, peaks corresponding to carbon (C) and gold (Au) were seen at 0.28 keV and 2.1 keV respectively which is an artifact from the sample preparation for SEM imaging. It can be observed phosphorus confirming the counter-ion used in Fig. 2A and C.

### Circular dichroism spectroscopy for secondary structure analysis of SP-A peptides

**Fig. 3:**
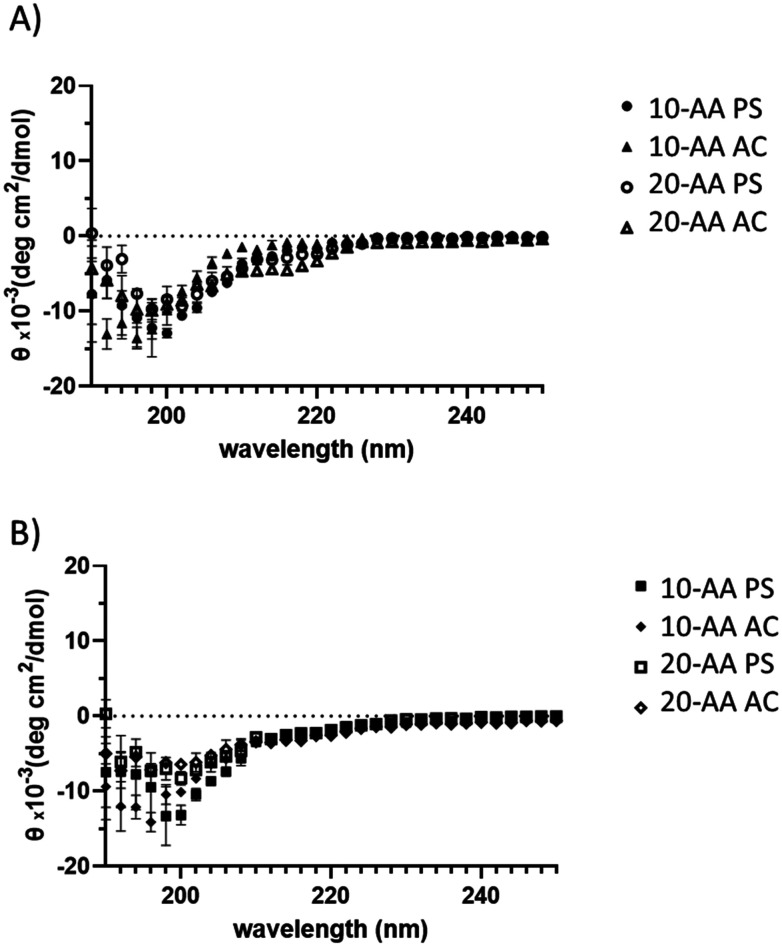
Circular dichroism (CD) of SP-A peptides at 2 different temperatures: (A) SP-A 10-AA and SP-A 20-AA at 25 °C (B) SP-A 10-AA and SP-A 20-AA at 37 °C. (*N* = 3).

The Circular Dichroism (CD) spectra for SP-A -10 AA and SP-A -20 AA peptides at different concentrations are shown in Fig. 3. The spectra confirm that SP-A 10-AA and SP-A 20-AA peptides predominantly assume a disordered conformation, consistent with their short sequence length, which limits the formation of stable secondary structures.^18^ No significant α-helical or β-sheet content was detected in either peptide, across all conditions tested (temperature and counterion variation). Although a negative peak around 202 nm is observed in SP-A 10, particularly in PS conditions, this does not indicate a well-defined secondary structure, but rather a typical signal of disordered peptides with minor transient conformations. Moreover, the absence of a clear 222 nm minimum and the lack of structural persistence across conditions confirm that SP-A 10-AA does not form a stable α-helix or β-sheet structure. This is further supported by molecular simulations, which demonstrate that the peptide remains predominantly disordered in solutions, although the longer sequence might be expected to stabilize some secondary structure, its CD spectra does not exhibit well-defined α-helical or β-sheet structures. Instead, its CD spectra features remain consistent with a random coil conformation, similar to SP-A 10-AA, across both 25 °C and 37 °C.

Additionally, temperature does not significantly alter peptide conformation, nor does the choice of counterion lead to major structural stabilization. This suggests that ionic interactions alone are not sufficient to induce secondary structure formation in these short peptides. These findings are consistent with studies on disordered peptides, where local sequence propensity and environmental conditions can influence transient conformations but do not necessarily stabilize well-defined structures.^10^

### Differential scanning calorimetry for thermal stability and phase transition analysis

**Fig. 4:**
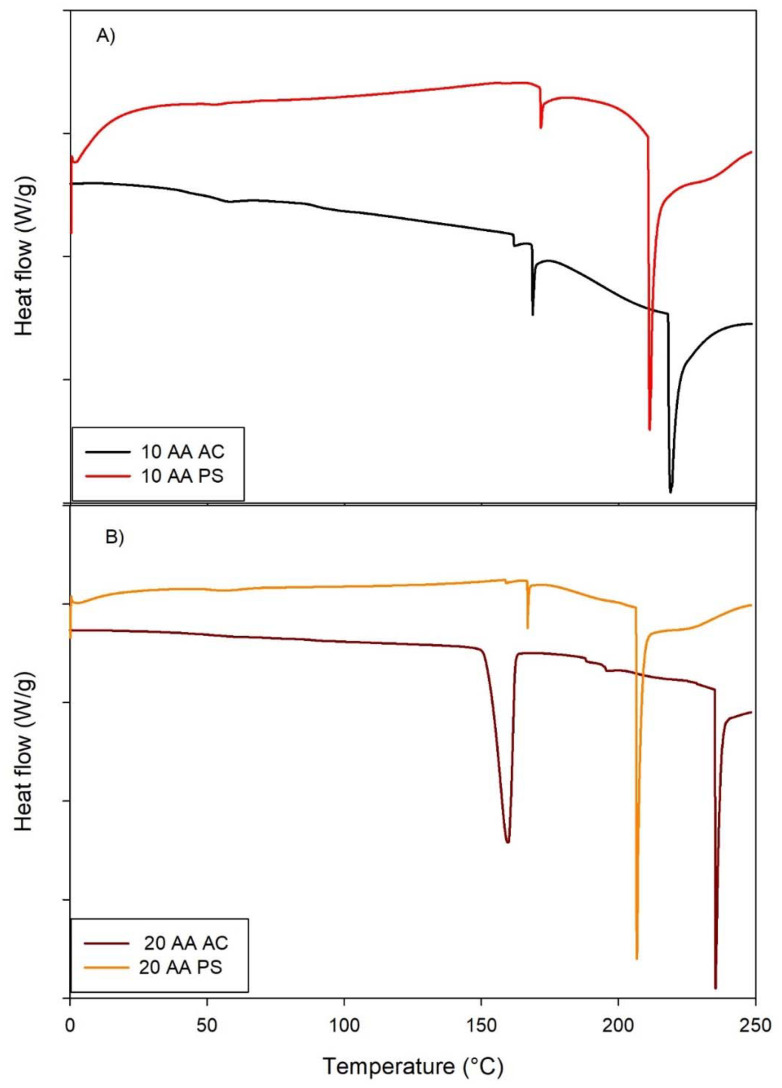
Differential scanning calorimetry (DSC) thermograms of SP-A: (A) 10-AA and SP-A (B) 20-AA Peptides. (Exothermic peak is up. Endothermic peak is down). (*N* = 3).

The DSC thermograms of SP-A 10-AA and SP-A -20 AA peptides in PS and AC salt forms exhibit unique thermal responses, indicating structural and stability variations (Fig. 4). The 10-AA AC peptide exhibits a broad endothermic transition about 150 °C, indicating a phase transition or unfolding event, but the 10-AA PS peptide has a sharper endothermic peak around 200 °C, indicating a more defined thermal transition. The AC and PS forms of the 20-AA peptides exhibit several endothermic transitions, with the AC form showing a prominent peak around 200 °C and the PS form transitioning at both 150 °C and 200 °C. The DSC thermogram of each peptide shows a characteristic endothermic peak at 168 °C, 171 °C, 158 °C and 166 °C for SP-A 10-AA AC, PS and 20 AC, PS, respectively, which is suggestive of their respective melting temperatures (*T*_m_). The enthalpy value (Δ*H*) was measured to be 1.96 J g^−1^, 3.08 J g^−1^, 1.08 J g^−1^, and 1.63 J g^−1^ for SP-A 10-AA -mer AC, SP-A 10-AA PS, SP-A 20-AA AC, and SP-A PS, respectively. This thermodynamic parameter indicates the heat required to break the molecular bonds that will lead to melting of the compound eventually.

### X-ray powder diffraction for crystallinity and structural analysis

**Fig. 5:**
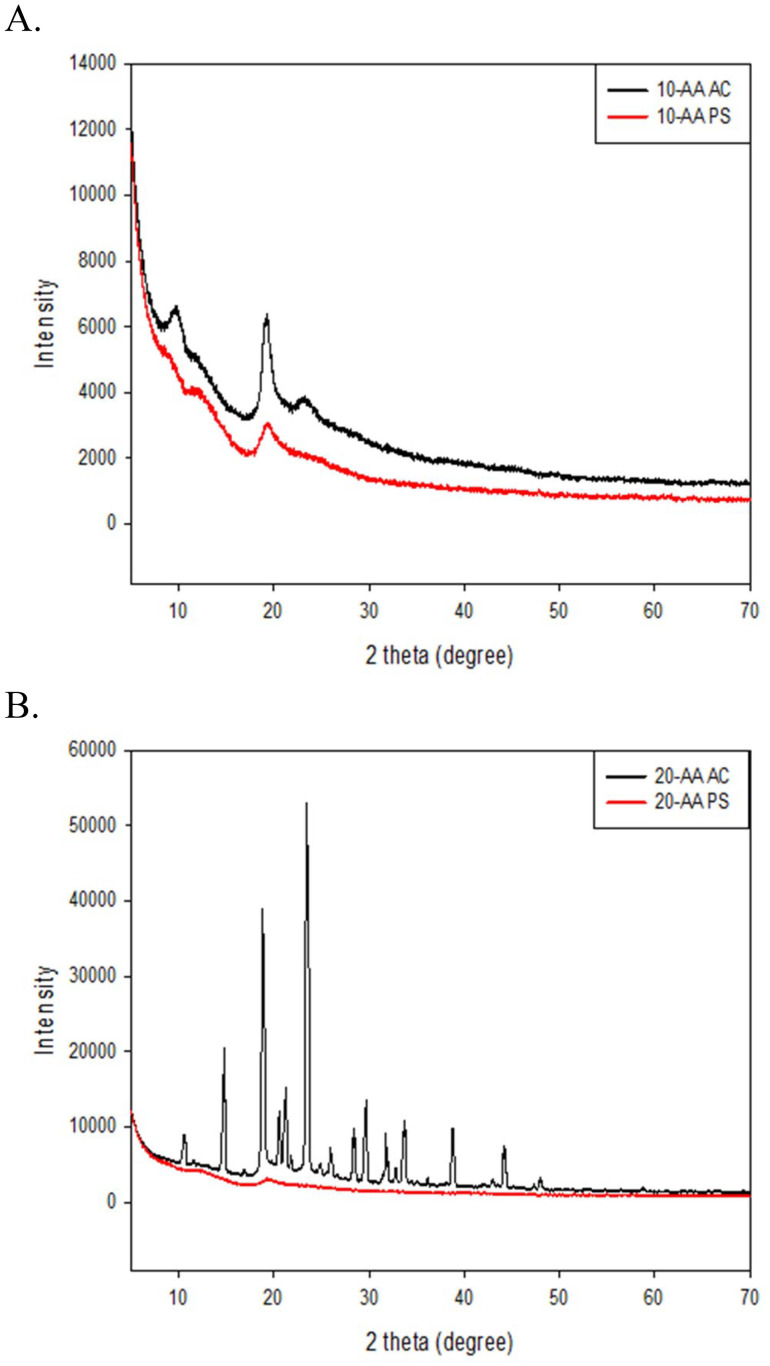
X-ray powder diffraction (XRPD) of SP-A peptides: (A) SP-A 10-AA AC and SP-A 10-AA PS; and (B) SP-A 20-AA AC and SP-A 20-AA PS.

The structural properties of the two SP-A peptides salts were studied using XRPD. The main diffraction peaks observed for both the peptides are shown in Fig. 5. In solid state, both peptides exhibited a peak around 19.5° 2*θ* at room temperature. Hence, the peak seen at 19.5° 2*θ* indicates that the peptides can have a degree of order, similar to other peptides reported.^11^ The 20-AA AC peptide showed the sharp diffraction peak pattern of a highly crystalline powders.

### Attenuated total reflectance-Fourier transform infrared spectroscopy (ATR-FTIR) for structural and chemical analysis

IR spectrum is used to detect and identify the chemical functional groups present in the compound which can be used as molecular fingerprint. The several molecular vibrations in a macromolecule leads to absorption peaks, hence it is difficult to assign every single peak to its corresponding bond. However, there are some characteristic peaks that are used to identify and study the secondary structure of proteins and peptides. Amide linkage in peptide/protein molecules are very active in IR mode which enables the characterization of biomolecules including a strong band at 1,600–1690 cm^−1^ (amide I), 1560–1540 cm^−1^ for amide II and 1370–1350 cm^−1^ for C–N.

**Fig. 6:**
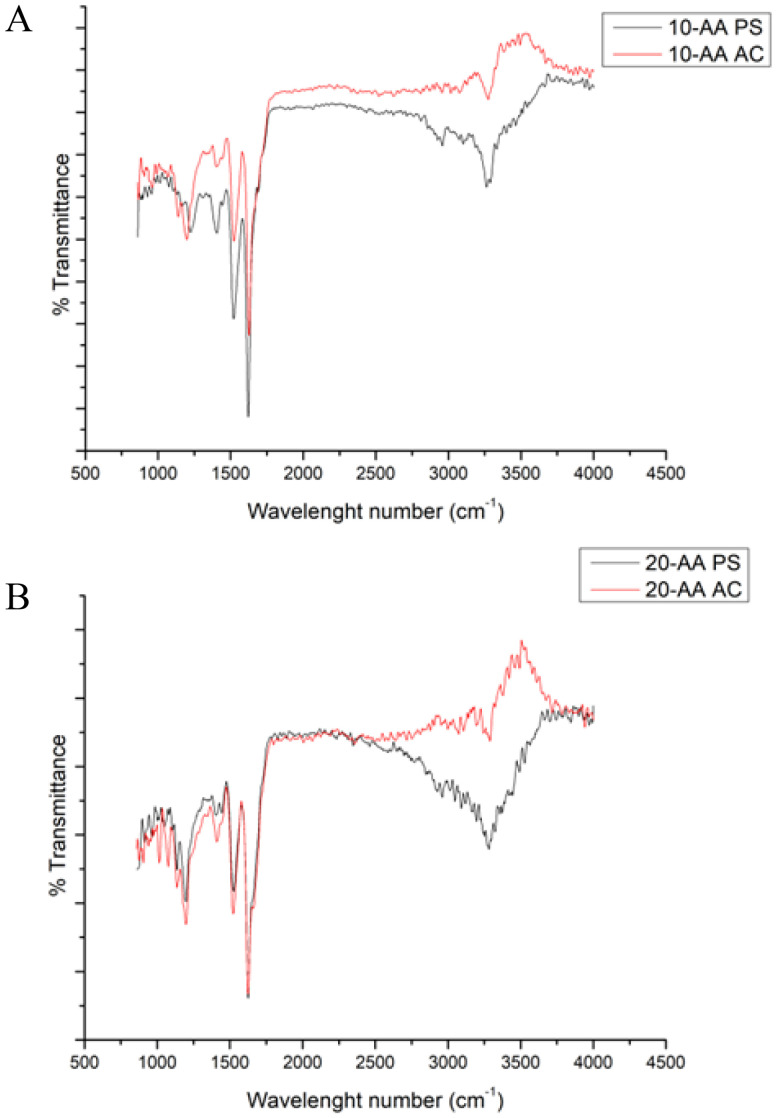
ATR-FTIR spectra of SP-A peptides: (A) SP-A 10-AA; and (B) SP-A 20-AA Peptides. (*N* = 3).

The ATR-FTIR spectra of SP-A 10 and SP-A 20-AA peptides in their AC and PS salt forms were analyzed in the amide I region (1600–1700 cm^−1^) to determine their secondary structure (Fig. 6). All spectras exhibit a predominant band centered at 1624–1627 cm^−1^, whose intensity and width suggest an extended or disordered structural conformation. Spectral deconvolution indicates that this signal accounts for 47% to 86% of the total structural content, with the highest proportion observed in SP-A 10 AC (86.13%), suggesting a strong tendency toward aggregation. Although the 1624–1627 cm^−1^ region is dominant, its broad profile suggests that these signals may arise from not only β-sheet structures but also aggregated disordered conformations.^19^ The higher proportion of signal in the 1650–1661 cm^−1^ region in SP-A 20-AA PS and SP-A 20-AA AC indicates increased structural flexibility, possibly influenced by the phosphate counterion.^20^

### Raman spectroscopy for molecular fingerprinting and structural characterization

**Fig. 7:**
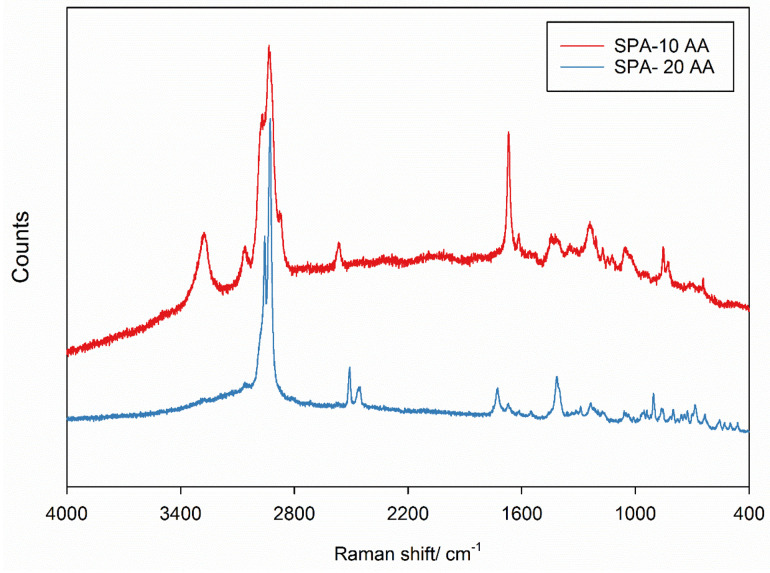
Confocal Raman spectra of SP-A 10-AA and SP-A 20-AA peptides.

The Raman spectra of SP-A -10 AA and SP-A -20 AA peptides show unique vibrational characteristics, indicating structural changes (Fig. 7). The peaks in the 2800–3000 cm^−1^ area, which indicate C–H stretching vibrations, are stronger for SP-A 10-AA. This indicates a higher concentration or a different environment for aliphatic chains in this peptide. The peaks in the 1600–1700 cm^−1^ area belong to amide I vibrations, linked to the peptide backbone's secondary structure, with significant intensity changes between the peptides. SP-A -20 AA exhibits distinct peaks in the 800–1200 cm^−1^ range, representing C–C, C–N, and N–H bending vibrations, and in the 1200–1400 cm^−1^ area, representing CH_2_ and CH_3_ bending modes. These spectrum discrepancies imply variations in backbone conformation and side-chain interactions, which may reflect differences in secondary structures like alpha-helices, beta-sheets, or random coils.^21^

### Zeta potential measurement for peptides charge

The zeta potential (*ζ*) results are shown in Table 1. Both the SP-A 10-AA and 20-AA peptides show net negative surface charge under these conditions, as expected from their amino acid sequence. The zeta potential values more positive than −20 mV in <30 mM diluent indicating a relatively low anionic surface charge.^22^ The 10-AA become less net anionic with increasing salt concentration for both salts. The SP-A 20-AA PS peptide became more net anionic with increasing salt concentrations, *ζ* of −20 mV at 0.5× PBS and NS. SP-A 20-AA AC peptide showed the same trend as the SP-A 10-AA peptide. This cutoff has been shown to correlate with aggregation, with lower absolute zeta being more likely to aggregate when charge is the predominant driver of aggregation.^22,23^

**Table 1:** Zeta potential of SP-A peptides in different solvents at 25 °C (*N* = 3, mean ± standard deviation)

Sample	*ζ* (mV)					
	Phosphate buffered saline			Normal saline		
	0.1×	0.2×	0.5×	0.1×	0.2×	0.5×
SP-A 10-AA PS	−7.7 ± 0.25	−9.6 ± 2.09	−2.7 ± 0.84	−6.5 ± 0.81	−7.4 ± 1.66	−3.5 ± 1.34
SP-A 20-AA PS	−5.5 ± 0.56	−11.1 ± 0.38	−21.4 ± 1.42	−3.1 ± 1.03	−1.6 ± 1.6	−18.4 ± 4.76
SP-A 10-AA AC	−7.9 ± 1.5	−3.9 ± 1.9	−3.7 ± 1.06	−5.6 ± 1.2	−5.36 ± 0.25	−4.9 ± 0.48
SP-A 20-AA AC	−9.0 ± 0.16	−2.8 ± 1.52	−5.2 ± 1.01	−7.8 ± 0.62	−4.91 ± 0.63	−3.0 ± 0.75

### Coulometric Karl Fischer titration for precise moisture content analysis

Karl Fisher titration is performed to measure the residual water content in the powder. Residual water content (Table 2) of the two peptides were measured to be 6.31 ± 1.30% and 3.12 ± 0.36% w/w for SP-A 10-AA PS and 20-AA PS peptides, respectively. On the other hand, SP-A 10-AA AC and 20-AA AC peptide powders show residual water contents of 4.6 ± 1.12% w/w and 1.9 ± 0.21% w/w, respectively.

**Table 2:** Residual water content by coulometric Karl Fischer titration (*N* = 3, mean ± standard deviation)

SP-A peptide	Residual water content (% w/w)
10-AA PS	6.31 ± 1.30
10-AA AC	4.6 ± 1.12
20-AA PS	3.12 ± 0.36
20-AA AC	1.9 ± 0.21

### *In vitro* solubility assessment for peptide formulation and bioavailability

The *in vitro* solubility results are presented in Table 3. The solubility of the SP-A 10-AA PS peptide was measured to be 12.2 ± 0.05 mg mL^−1^ in MQ water, 1.81 ± 0.02 mg mL^−1^ in PBS, 2.06 ± 0.03 mg mL^−1^ in EtOH, and 4.25 ± 0.01 mg mL^−1^ in methanol. The SP-A 10-AA AC showed less solubility in aqueous solutions *i.e.* 0.42 ± 0.040 mg mL^−1^ in MQ water, 1.16 ± 0.01 mg mL^−1^ in PBS, 0.04 ± 0.01 mg mL^−1^ in EtOH, and 0.01 ± 0.0003 mg mL^−1^ in methanol.

**Table 3:** Solubility at ambient room temperature of SP-A peptides in different solvents (*N* = 3, mean ± standard deviation)

Solvent	10-AA PS solubility (mg mL^−1^)	10-AA AC solubility (mg mL^−1^)	20-AA PS solubility (mg mL^−1^)	20-AA AC solubility (mg mL^−1^)
MilliQ water	12.2 ± 0.05	0.42 ± 0.040	10.36 ± 0.034	0.08 ± 0.029
PBS (pH 7.4)	1.81 ± 0.02	1.16 ± 0.01	0.47 ± 0.004	0.30 ± 0.01
Absolute ethanol	2.06 ± 0.03	0.04 ± 0.01	0.11 ± 0.05	0.02 ± 0.02
Methanol	4.25 ± 0.01	0.01 ± 0.0003	1.45 ± 0.02	0.02 ± 0.005

For the solubility between SP-A 20-AA PS and AC different salts, same trend was observed. SP-A 20-AA PS solubility was measured to be 10.36 ± 0.034 mg mL^−1^ in MQ water, 0.47 ± 0.004 mg mL^−1^ in PBS, and 0.11 ± 0.05 mg mL^−1^ in EtOH 1.45 ± 0.02 mg mL^−1^ in methanol. The AC 20-AA SP-A salt shows very different solubility properties, namely, 0.08 ± 0.029 mg mL^−1^ in MQ water, 0.30 ± 0.01 mg mL^−1^ in PBS, 0.02 ± 0.02 mg mL^−1^ in EtOH, and 0.02 ± 0.005 mg mL^−1^ in methanol. The solubility properties demonstrate the effect of the counterion type on the solubility value independent of the same length-peptide.

### Octanol/water partition coefficient for lipophilicity and drug permeability assessment

The octanol/water partition coefficient (log *P*) studies of the SP-A 10-AA and SP-A 20-AA peptides in PS and AC salt forms indicate unique hydrophilic and hydrophobic qualities that are critical for their biological activity. As shown in Table 4, the 10-AA peptide in both salt forms is more soluble in water than in octanol, with log *P* values of −1.79 PS and −1.07 AC, indicating a hydrophilic character. The 20-AA peptide has hydrophilic properties, with log *P* values of −0.81 PS and −0.91 AC, yet it is somewhat more hydrophobic than the 10-AA peptide.

**Table 4:** Octanol/water partition coefficient and log *P* of SP-A 10-AA and SP-A 20-AA peptide at *T* = 37 °C (*N* = 3, mean ± standard deviation)

Solvent	10-AA PS	10-AA AC	20-AA PS	20-AA AC
MilliQ water	1.12 ± 0.5 mg mL^−1^	2.06 ± 0.03 mg mL^−1^	1.49 ± 0.015 mg mL^−1^	0.91 ± 0.01 mg mL^−1^
Octanol	0.47 ± 0.004 mg mL^−1^	0.17 ± 0.02 mg mL^−1^	0.22 ± 0.009 mg mL^−1^	0.11 ± 0.01 mg mL^−1^
Log *P*	−1.79 ± 0.4	−1.07 ± 0.2	−0.81 ± 0.3	−0.91 ± 0.2

### High performance liquid chromatography

**Fig. 8:**
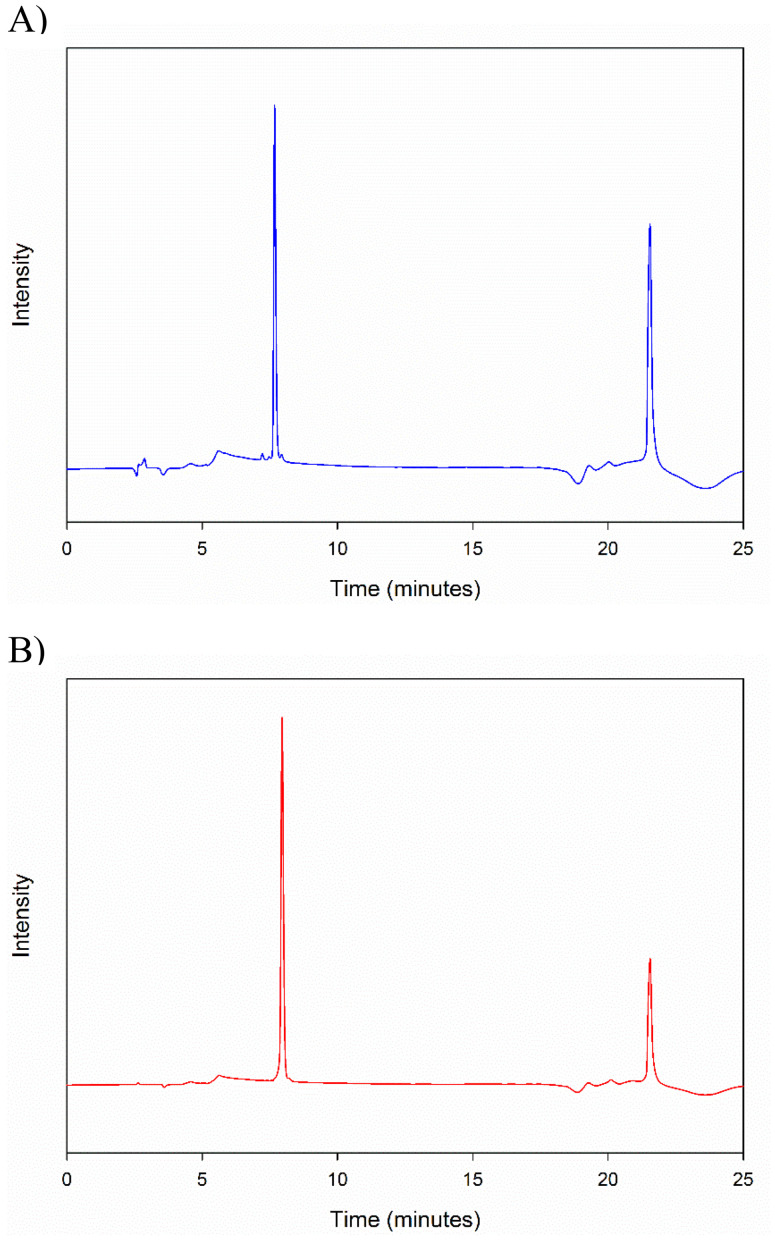
High performance liquid chromatography of SP-A Peptides: (A) SP-A 10-AA; and (B) SP-A 20-AA.

The HPLC chromatogram of peptides 10-AA and 20-AA are shown in Fig. 8. The retention time of 10-AA peptide was around 7.6 minutes and 20-AA peptide was around 7.9 minutes. The peak seen around 22 minutes is probably from the change in polarity of the mobile phase, since this peak was also seen in blank solvent injection.

### *In vitro* cell viability assessment for biocompatibility and cytotoxicity evaluation

**Fig. 9:**
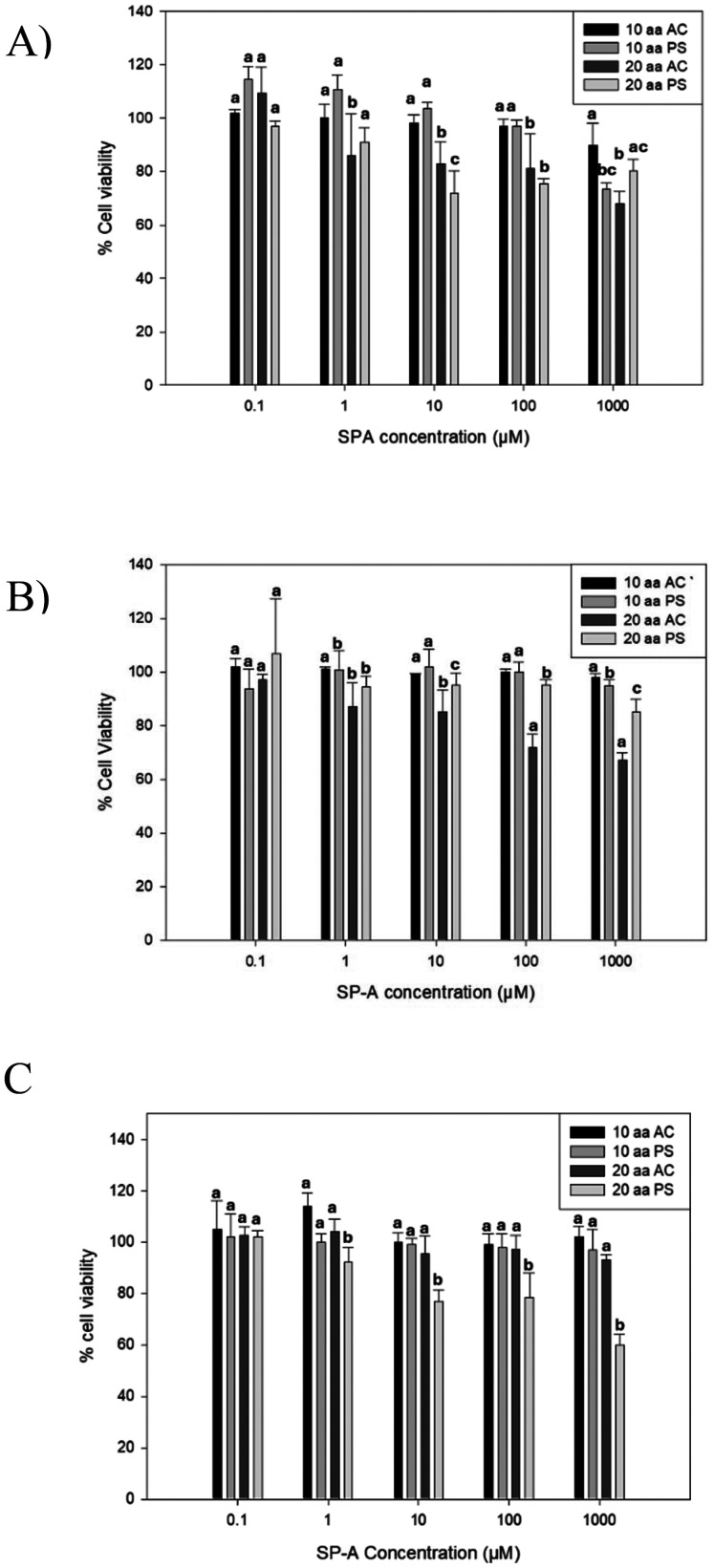
*In vitro* cell viability of human lung cells at various doses of SP-A 10-AA and SP-A 20-AA peptides: (A) H441; (B) A549; and (C) H358. (*N* = 6). Significant differences are indicated by different letters (*p* < 0.05).

The *in vitro* cell viability experiments performed with different pulmonary cell lines exhibits the cytotoxic effect of the four peptides in Fig. 9. The A549 and H441 cell lines showed no cytotoxicity effect but the 20-AA AC by affecting the relative cell viability for SP-A concentration higher than 100 μM. For H38 cell line, the only peptide that had a cell viability effect was the 20-AA PS at its highest concentration. The results indicate that A549 and H441 cells maintained high viability across all peptide treatments, with no statistically significant reductions, except for 20-AA AC, which significantly reduced cell viability at concentrations above 100 μM (*p* < 0.05). In contrast, the H358 cell line exhibited a statistically significant decrease in viability only with 20-AA PS at 1000 μM (*p* < 0.05), suggesting a selective cytotoxic response in this cell line. Most peptide formulations exhibit no significant cytotoxic effects, supporting their biocompatibility for potential pulmonary applications and that is a concentration-dependent cytotoxicity in 20-AA AC and 20-AA PS highlights the importance of formulation selection for optimizing peptide-based inhalation therapies.

### *In vitro* transepithelial electrical resistance (TEER) measurement for barrier integrity and permeability assessment

**Fig. 10:**
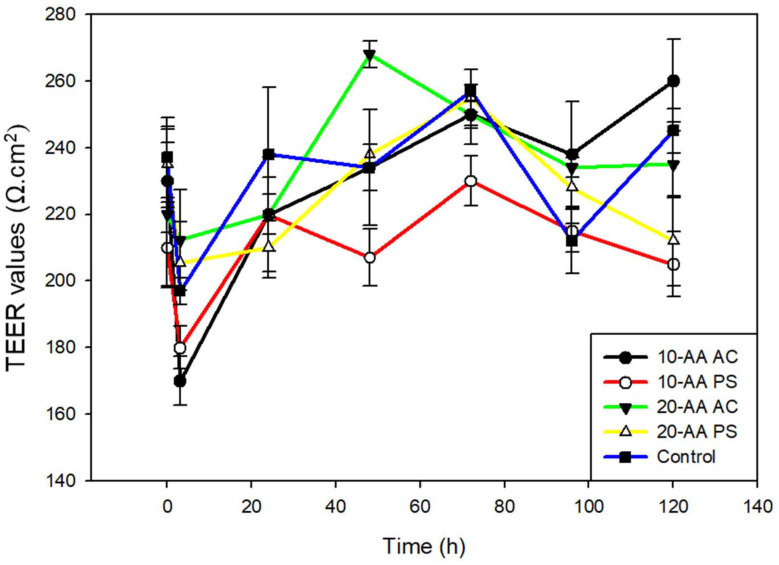
Transepithelial electrical resistance (TEER) following dosing of SP-A 10-AA and SP-A 20-AA peptides on H441 human pulmonary cells at the air–liquid interface (ALI). (*N* = 3).

The barrier properties of the H-441 cell line were evaluated at the air–liquid interface (ALI). The TEER values of H-441 cell lines treated with both salts of 10-AA and 20-AA peptides showed similar patterns in Fig. 10. The electrical resistance of the cells treated with peptides were similar to the control cells which was treated with the RPMI media.

### Molecular dynamics simulation for structural stability and conformational analysis of SP-A peptides

The Fig. 12 presents the structural evolution and secondary structure stability of SP-A 10 and SP-A 20-AA peptides over a 10 ns MD simulation. To further understand secondary structure retention and dynamic flexibility, we assessed the conformational stability of the 10-AA and 20-AA peptides throughout a 10 ns simulation. Fig. 12 presents the structural evolution and secondary structure stability of SP-A 10-AA and SP-A 20-AA peptides over a 10 ns MD simulation. Fig. 12A–D show the initial (0 ns) and final (10 ns) conformations of each peptide, highlighting structural changes, in SP-A 10-AA, a progressive loss of α-helical content is observed after 5 ns, indicating structural flexibility and instability, in contrast, SP-A 20-AA does not exhibit any α-helical secondary structure, maintaining a predominantly disordered conformation throughout the simulation. The Fig. 12E and F shows the evolution of α-helical content over simulation time, with SP-A 10-AA gradually decreasing while SP-A 20-AA remains as random coil structure. MD simulations provided insights into the structural stability and conformational evolution of the SP-A 10-AA and SP-A 20-AA peptides over a 10 ns simulation. The results revealed significant differences in their ability to maintain secondary structure.

## Discussion

The objective of this study was to characterize the physicochemical properties and *in vitro* cellular effects of SP-A peptides, particularly their biocompatibility and interaction with lung epithelial cells. The *in vitro* results demonstrated that SP-A peptides maintain high cell viability, do not significantly alter epithelial barrier integrity, and do not increase membrane permeability. These findings indicate that the peptides are well tolerated in lung models, reinforcing their potential as safe and effective candidates for inhaled therapeutic applications. It is has been identified that the 10-AA derived from carbohydrate recognition domain of SP-A2 has activity similar to the full length oligomeric protein in treating inflammatory aspects of asthma.^4,24^ The use of small SP-A-derived peptides offers several advantages over full-length SP-A for therapeutic applications, including enhanced stability, greater bioavailability, and improved formulation feasibility, such as drug delivery. Furthermore, XRD examination shows that the 20-AA peptide has higher crystallinity, indicating a more organized structure equivalent to that found in full-length SP-A. The DSC study also confirms this by revealing increased heat stability, which is critical for retaining structural integrity during inhalation administration.

Additionally, SP-A peptides presenting the CRD domain have been demonstrated to retain functional activity, as seen in previous studies by Kraft and Ledford.^25^ showing that SP-A peptides covering the CRD domain maintain bioactivity while successfully reducing airway hyperresponsiveness and inflammation in preclinical asthma models. Our peptides’ structural stability, as validated by XRD, CD, and DSC studies, strengthens their capacity to modulate surfactant activity and immunological responses in the same way that full-length SP-A work. Furthermore, given the association between SP-A deficiency and increased airway inflammation, these peptides may be potential candidates for inhalation therapy to restore SP-A-like function in respiratory disorders such as asthma.

Hence, the goal is to develop this peptide into a novel therapy to treat asthma that will be delivered locally to the respiratory tract. Currently, inhaled drugs targeted directly to the lungs has become the preferred route of drug delivery used clinically as successful pharmaceutical products in many respiratory diseases. Aerosol therapeutic drug development is a complex process where the particles or droplets need to be of sufficient size distribution for targeted delivery.^26^ Consequently, several novel technologies are involved in development and optimization of these inhaled aerosols that can expose the drug compound to different conditions with respect to temperature, pressure, pH *etc*. Therefore, it is imperative to characterize the therapeutic peptides to understand the important biophysical and chemical properties such as phase transition, thermal behavior, and secondary structure that contribute to its development.

Microscopic imaging enables us to see the secondary structure, the three-dimensional structure of the peptide, and the self-assembly or aggregation of the peptide, if any. Hence, this observation in unprocessed peptides will help understand the changes that the peptide may undergo during formulation development. Similarly, techniques such as DSC, XRPD, ATR-FTIR, Raman spectroscopy are used to characterize the peptide before and after processing to study the physical or chemical changes that might occur to the peptide. This information is key for the development of therapeutic peptides since it may affect the stability and biological activity of the peptide. The elemental characterization technique, EDX, determined that the peptides were mainly composed of carbon, oxygen, and nitrogen, but elements such as sulfur were found in both 10-AA and 20-AA peptides. The source of sulfur atoms will be the cysteine and methionine amino acids that are part of the primary structure of both peptides.

Circular Dichroism is the commonly used light absorption technique to detect and determine the secondary structure of peptides and proteins. The CD spectra of the two SP-A peptides in solution are shown in Fig. 3. The length of the amino acid chain in peptides has a significant impact on their secondary structure and stability, as shown for SP-A -10 AA and SP-A 20-AA peptides. The CD spectra confirm that the SP-A 10-AA and SP-A 20-AA peptides predominantly adopt a disordered conformation, as expected due to their short length, which prevents the formation of stable secondary structures.^27^ Our analysis shows that SP-A 10-AA at both temperatures (25 °C and the 37 °C) exhibits a characteristic profile of disordered peptides, with minimal to no α-helical or β-sheet content. SP-A 10-AA exhibits a modest negative ellipticity about 202 nm, indicating that a small portion of its population may briefly adopt an α-helical shape. One possible explanation for this behavior is electrostatic interactions between the counterion and the peptide, which may temporarily reduce backbone solvation, allowing short-range hydrogen bonding to persist.^28^ While this helicity is marginal and does not constitute a stable secondary structure, it aligns with prior studies in short peptides, where local sequence propensity and ionic interactions can induce partial helicity in otherwise disordered peptides.^18^

Trifluoroethanol (TFE) has been shown to reduce backbone solvation and promote intramolecular hydrogen bonding in disordered peptides, leading to α-helical structures.^29^ Given that SP-A 10-AA and 20-AA were derived from a portion of the domain of SP-A carbohydrate recognition, it was expected that they would exhibit some structural and physical characteristics comparable to those of the natural protein. This finding is consistent with previous research on peptides generated from proteins that perform comparable biological roles.^30^ CD analysis is an important tool in the creation and evaluation of therapeutic peptides because it reveals information about their secondary structure in solution and their capacity to preserve structural integrity under physiological conditions.

ATR-FTIR spectroscopy was used to analyze the secondary structure of SP-A peptides, focusing on the Amide I region (1600–1700 cm^−1^). Deconvolution of the Amide I region in the ATR-FTIR spectra revealed a dominant band around 1624–1627 cm^−1^, which is best attributed to aggregated random coil conformations. This assignment is strongly supported by CD spectra, which indicate a predominant random coil signature, and MD simulations, which show a progressive loss of secondary structure, with the peptides transitioning into fully disordered states over time. The broad nature of this FTIR band suggests the presence of strong inter-chain hydrogen bonding within disordered aggregates, a feature commonly observed in peptides that lack stable secondary structures but tend to self-associate. The combination of these findings—CD spectra showing a random coil signature, MD simulations revealing structural destabilization, and ATR-FTIR indicating aggregated disordered conformations.^31–33^

ATR-FTIR provided greater sensitivity to structural changes than CD spectroscopy, but the combined data indicate that the observed secondary structure should be interpreted as aggregated disordered structures rather than a defined secondary structure, which has important implications for formulation stability and peptide interactions.^34,35^

**Fig. 11:**
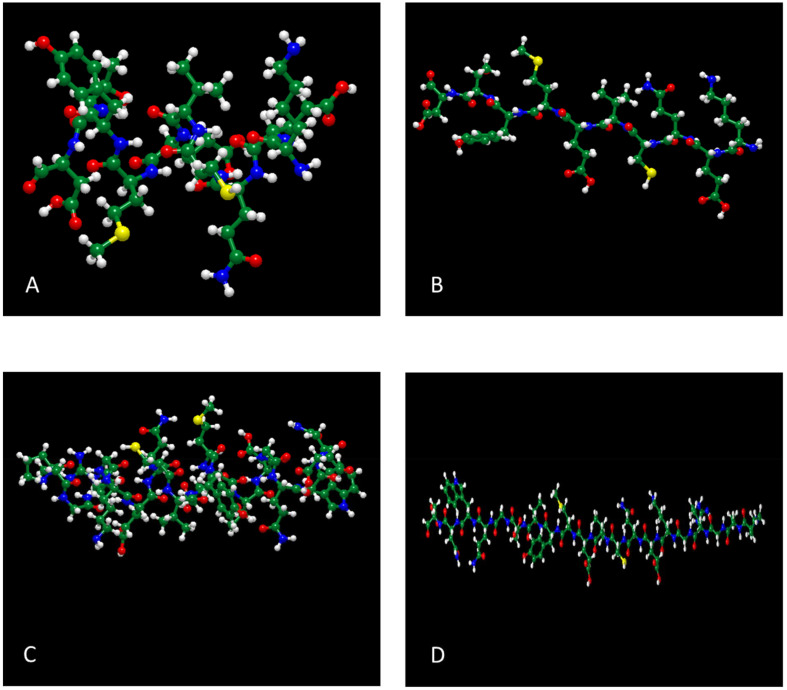
Energy minimized 3-D models of SPA peptides by molecular modeling (Molecular Modeling Pro® Software) for: (A) SPA 10-AA; (B) SPA 10-AA energy minimized; (C) SPA 20-AA; and (D) SPA 20-AA energy minimized.

Using energy minimization in molecular modeling software programs (ChemSite Pro® and Molecular Modeling Pro®), Fig. 11 shows that both the α-helix and β-sheet secondary structure conformations are thermodynamically stable. Calculations from Fig. 11 for 10-AA peptide, the α-helix structure (Fig. 11A) reveals that the distance, *d*, from lysine 1 to Asp 10 is 18.53 Å, whereas β-sheet (Fig. 11B) calculations shows that the *d* is 26.72 Å *i.e.* predictive longer length for the β-sheet conformation. For the 20-AA peptide, α-helix and β-sheet structures showed an intramolecular distance from Pro 1 to Aps 20 of 31.39 Å and 66.16 Å, respectively. After energy minimization for all peptide structures, the predicted total free energy was −130.24 kcal mol^−1^, −178.4 kcal mol^−1^, −65.05 kcal mol^−1^, and −7.4 kcal mol^−1^ for 10-AA α-helix, 20-AA α-helix, 10-AA β-sheet, and 20-AA β-sheet structures, respectively, suggesting that the α-helix conformation is more stable in these minimized structures. However, CD spectra indicate that the peptides predominantly adopt a random coil conformation rather than a well-defined α-helix in the solid state, suggesting that these secondary structures are not maintained in experimental conditions. These energy minimization results provide a static representation of potential conformations but do not account for structural fluctuations in solution, which are better captured through MD simulations.

Hence, this suggests that the α-helix structure is favorable in the peptide, which agrees with the CD spectra, which show the presence of a marginally α-helix secondary structure in a solid state. These energy minimization results provide a static representation of the peptides’ initial conformations. However, they do not account for structural fluctuations in solution, which are better captured through MD simulations.

**Fig. 12:**
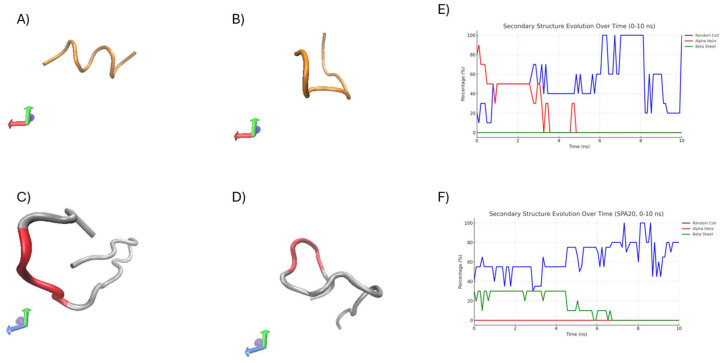
Structural evolution and α-helical stability of SP-A 10-AA and SP-A 20-AA peptides from MD simulations. MD simulation results of SP-A 10-AA and SP-A 20-AA. (A–D) Structural snapshots at 0 ns and 10 ns. (E and F) Secondary structure evolution over time.

MD simulations provide important information about the structural behavior and stability of peptides under physiological settings. In agreement with the small negative ellipticity observed at 202 nm in the CD spectra, MD reveals that SP-A 10-AA exhibits transient α-helical structure, though it remains unstable and does not persist throughout the simulation (Fig. 12). This aligns with prior studies suggesting that short peptides can form momentary helices due to sequence propensity and ionic interactions, but these do not constitute stable secondary structures. Notably, SP-A 20-AA does not exhibit significant helicity in either CD or MD, reinforcing its inherently disordered nature. The MD data also reveals the presence of transient β-sheet formation in SP-A 20-AA, which was not evident in the CD spectra, likely due to its low population and instability over time.

The anionic 10-AA PS and 20-AA PS salt peptides showed more hydrophilic behavior than their AC salt form with good solubility in various aqueous solvents as evident from Table 3. This is consistent with the known hydrophobic property of the AC salt. Both peptide salts had limited solubility in common alcohols. The octanol/water partition study also suggests that the peptides have higher affinity to the aqueous phase than the organic phase. The predicted log *P* of 10-AA PS and 20-AA PS peptides were −1.79 ± 0.4 and −0.81 ± 0.2, respectively. Log *P* values for the AC salts were 1.07 ± 0.3 and −0.91 ± 0.2 for 10-AA AC and 20-AA AC, respectively. The negative log *P* value agrees with the very low solubility shown in less polar solvents as ethanol and methanol in Table 4. The AC versions of both peptides have better water solubility and smaller negative log *P* values than their PS counterparts, implying that the AC salt promotes hydrophilicity. These changes in solubility and partitioning qualities affect the peptides’ bioavailability and cellular uptake, both of which are crucial for their efficacy in pulmonary applications, emphasizing the need of optimizing salt forms for therapeutic usage. The AC versions of both peptides had a higher solubility in water and consequently lower negative log *P* values, demonstrating that AC salts increase the peptides’ hydrophilicity. As a result, the salt form used can have a direct impact on peptide bioavailability, cellular uptake, and overall therapeutic efficacy.

A peptides solubility is regulated by a variety of physicochemical characteristics, including its net charge at physiological pH, hydrophilic–hydrophobic balance, and the presence of particular counterions. We investigated the solubility of SP-A peptides in relation to their estimated pI and experimentally measured log *P* values. The estimated pI values for SP-A 10 and SP-A 20-AA peptides were 3.69 and 3.93, respectively, revealing their charge distribution in aqueous environments and implying that their solubility is significantly based on the solution pH relative to their pI. Peptides have lower solubility near their pI due to reduced electrostatic repulsions, which promote aggregation and precipitation, but they are more soluble when carrying a net charge at pH values above or below their pI.^35^

Our experimental solubility findings align with theoretical predictions, demonstrating that peptide solubility is significantly higher in conditions where they carry a net charge, confirming the electrostatic contribution to solubility. The presence of PS and AC counterions influenced hydration shells and electrostatic interactions, leading to ion-specific effects on solubility.^34^ These ion-specific solubility effects are particularly relevant for peptide formulation, as salt selection plays a critical role in preventing aggregation and enhancing shelf-life stability. Understanding these counterion-dependent solubility trends provides valuable insights for optimizing peptide formulations, particularly for pharmaceutical and biotechnological applications.

The log *P* parameter describes how peptides partition between aqueous and lipidic environments, affecting their interaction with biological membranes and potential for drug administration.^36^ A balanced hydrophilic-lipophilic profile is essential for successful dissolving in aqueous settings while retaining the ability to interact with cell membranes, especially in inhalable formulations that need diffusion across the lung epithelium. Our findings show that SP-A 10-AA and SP-A 20-AA peptides have distinct hydrophilic and hydrophobic properties, with log *P* values indicating that they can preserve solubility while retaining membrane affinity, which is critical for pulmonary delivery and therapeutic efficacy.^37^

The formulation and bioavailability of therapeutic peptides depend largely on their solubility and partitioning characteristics.^38^ According to our research, the peptide AC salt is more soluble in water than its PS version due probably to AC ions having weaker ionic interactions and less kosmotropic effects.^39^ The PS salt, on the other hand, has somewhat reduced solubility, which could be explained by more robust electrostatic interactions that encourage aggregation. Furthermore, log *P* research indicates that PS may promote interactions with lipid membranes, which could affect absorption and bioactivity, whereas AC prefer hydrophilic environments, improving systemic bioavailability in aqueous formulations.^20^ These results highlight how crucial counter-ion selection is when refining peptide formulations for certain therapeutic applications.

The Δ*H* value of 20-AA is higher than the 10-AA due to the increased aa length that requires more energy to break the intermolecular bonds before melting. No other transitions were observed before the melting transition indicating that these peptides are thermodynamically stable at room and biological temperatures.^40^ The longer chain of the 20-AA peptide results in more complex thermal behavior, which could be attributed to higher intramolecular interaction and structural stability. AC forms had larger and lower temperature transitions than PS forms, indicating that the salts influence peptide stability and interactions differently.

The high melting temperatures observed in the DSC thermograms suggest that intermolecular interactions, rather than a traditional folding–unfolding event, are the primary cause of the thermal changes of the SP-A 10-AA and SP-A 20-AA peptides. Strong peptide-peptide interactions and potential dehydration effects are indicated by the observed transitions, although it is not likely that these peptides would form highly stable secondary structures due to their short length. Further evidence that counterion-specific electrostatic interactions support thermal stability is provided by the differences between phosphate and acetate salts, with phosphate perhaps promoting aggregation or intermolecular hydrogen bonding. These results concur with earlier studies that found counterions affect the stability and self-assembly of peptides at higher temperatures.^41,42^ These findings are critical for understanding the peptides’ stability and applicability for therapeutic uses, as heat stability might affect storage, handling, and biological activity.

A peptide salt form can affect its solubility, thermal stability, and intermolecular interactions—all of which can be measured with DSC.^43^ The AC and PS salt versions of the peptides showed significant variations in heat stability, according to DSC themograms. The multivalent character of PS ions could mitigate charge repulsion and strengthen intra- and intermolecular contacts, as seen by the PS form's greater *T*_m_, which suggests improved electrostatic stability. The AC form, on the other hand, showed a somewhat lower *T*_m_, suggesting a less stable structure, possibly as a result of weaker ionic connections.^20^

XRPD is used to determine degree of crystallinity/non-crystallinity, polymorphs, and peptide secondary structures in the solid-state, as our group has reported.^11,14,44^For example, insulin is a protein macromolecule has been extensively characterized using crystallography and its several polymorphs of insulin were discovered using XRPD.^45^

Spectroscopic techniques such as FTIR and Raman can be used to characterize peptides and proteins due to the presence of the amide (peptide) bonds which is characteristic of the peptide/protein folding. The IR peaks seen in (Fig. 6) amide I (1700–1600 cm^−1^) region is primarily due to carbonyl groups in peptide (C=O stretching), while amide II (1500–1600 cm^−1^) and amide III (1220–1320 cm^−1^) regions consists of C–N stretching and N–H bending.^31^ These three amide regions are generally used to identify conformational changes of proteins and peptides. Complimenting this technique, Raman spectra (Fig. 7) also exhibits absorption bands in the three amide regions. The strongest peak seen in Raman, for both peptides, around 2900–3200 cm^−1^ is the C–H vibration in the peptides. Also, seen in the region of 2500–2600 cm^−1^ is the stretching vibration of sulfhydryl group.^46^ When using IR spectroscopy, a combination of the three amide regions are generally used to deduce the structure of the peptide/protein due to multiple overlapping from other bands. Among these, amide II band is the less sensitive and less frequently used for structural analysis,^47^ possibly due to its conformational sensitivity.^31,48^ Goormaghtigh *et al*. have reported that only three wavenumbers are useful in deducing the secondary structure of protein. The best wavenumbers identified to predict secondary structure are 1545 cm^−1^ for α-helix, 1656 cm^−1^ for β-sheet, 1677 cm^−1^ for β-turn and 1544 cm^−1^ for random structure. Absence of any of these bands in the spectra observed in this study suggests the complexity of characterizing peptide secondary structure in solid state. However, these spectroscopic data will serve as a qualitative tool to compare any changes that might occur to the peptides during formulation development or in the final drug product.^7^ In general, Raman and IR spectral data is processed using advanced chemometric analysis to determine the composition and secondary structure of protein/peptide and it is also common to correlate the structural determination obtained from vibrational spectroscopy with CD.

A dose–response cell viability study was performed on different pulmonary cell lines using the two SP-A peptides salts at different concentrations. The relative viability of the A549 cells remained high for the peptides at all tested concentrations. The 20-AA PS showed decreased viability at higher concentrations in the H-358 cell line and the 20-AA AC showed a reduction in viability at higher concentrations in H-441 cell lines. This *in vitro* cellular demonstrates that SP-A peptides are safe for pulmonary cell lines up to 100 μM. Fig. 9 shows cell viability values of cells treated with the peptides relative to control cells treated with cell growth media. Generally, a cell viability of 80% or higher is considered safe for *in vitro* lung studies. The TEER values observed in H441 human lung cells were similar to a previous study where the TEER value observed was a plateau.^17^ The TEER value observed in that study was that of naïve cells. A similar value seen in this study, where the H-441 cells were treated with the SP-A peptides demonstrate that the peptides didn't adversely affect the tight membrane interactions.

Their smaller size facilitates diffusion and deposition in the lung epithelium, making them ideal candidates for inhalation therapy. Additionally, peptide-based approaches are more cost-effective, scalable, and reduce immunogenicity concerns compared to recombinant full-length SP-A. These findings reinforce the relevance of SP-A-derived peptides in pulmonary drug development and highlight their advantages as therapeutic agents for respiratory diseases.

The high aqueous solubility of the PS peptides suggests that an aqueous-based liquid aerosol (solution) formulation will be a straightforward approach for the early phase testing of the safety and efficacy of the peptides *in vivo* models. However, highly hydrophilic compounds pose the risk of poor membrane permeability. For AC peptides a formulation using dry powder inhalers will be the more suitable pathway to produce a SP-A drug. Another key factor to consider is the chemical and conformational instability of proteins leading to denaturation and degradation in solutions.

Therefore, a liquid aerosol formulation of the SP-A peptides must consider excipients that will enhance the stability and permeation of the peptides. Alternately, the SP-A peptides AC can be developed in solid state as dry power aerosol which is less likely to affect chemical stability. In solid-state formulation chemical and conformational stability can be achieved by the right choice and concentration of excipients that can improve the physicochemical property. The most common approach is through glass stabilization, where the excipient and drug form a glassy amorphous structure (with limited molecular mobility) whose glass transition is higher than the peptide, rendering it stable over the usable temperature range. Other possible mechanism is through hydrogen bonding between the peptide chain and the excipient thus stabilizing the structure^49^ for longer period. The high temperature and drying cycles commonly encountered during the micronization process can affect the stability, in which case, cryoprotectants like sugar, amino acid,^50^ or organic salt can prevent the crystallization of protein that may occur during processing. Each physical state of the peptide has its own advantages and challenges. Hence, it is important to consider the possible options for the development of SP-A peptides formulation that can locally deliver to the lungs.

The 20-AA peptide's structural stability, as shown by CD spectra, is consistent with techniques employed in commercial therapeutic peptides like GLP-1 receptor agonists. The engineering of exenatide and liraglutide demonstrates the significance of secondary structure preservation in peptide drug design to preserve α-helical integrity in order to improve receptor affinity and bioactivity.^51^ As with these formulations, maintaining our peptides’ stability may be essential to their possible therapeutic use, impacting formulation techniques to increase bioavailability and preserve functional activity. Additionally, understanding the thermal properties of therapeutic peptides is essential for formulation development. The transition of liraglutide from a crystalline to an amorphous state during encapsulation highlights the impact of formulation processes on peptide stability. Similarly, the thermal stability of SP-A derived peptides will inform regarding storage conditions, delivery mechanisms, and potential excipient selection to maintain peptide integrity and therapeutic efficacy.

The observed transient helicity in SP-A 10-AA aligns with previous studies on short peptides, where local sequence composition and electrostatic interactions can induce partial helicity but fail to stabilize it over extended timescales. For example, studies on antimicrobial peptides, such as LL-37,^52^ have shown that helical stability is often context-dependent, requiring interactions with lipid membranes or other biomolecules to maintain secondary structure. The behavior of SP-A 10-AA may follow a similar paradigm, where the peptide exhibits α-helical character only in specific microenvironments, such as when bound to a receptor or embedded in a lipid bilayer.

## Conclusion

This study provides a comprehensive characterization of SP-A-derived peptides, integrating experimental and computational approaches to better understand their structural behavior, solubility, and biocompatibility, which are key factors for future formulation development. The findings reveal that SP-A 10-AA exhibits transient α-helicity (∼80–90% at 0 ns, decreasing over time), while SP-A 20-AA remains largely disordered throughout the simulation, suggesting that secondary structure stability is sequence-dependent and may influence formulation performance. Differences in solubility and partitioning behavior were also observed, with phosphate salts exhibiting higher aqueous solubility (log *P* ∼−2.5) compared to acetate salts (log *P* ∼−1.8), which could impact their dissolution rate and bioavailability in pulmonary delivery systems.

These insights emphasize the importance of physicochemical stability, secondary structure, and solubility in the design of peptide-based therapeutics. The observed differences in secondary structure and solubility among formulations suggest that selecting the appropriate salt form and excipients could enhance peptide stability, aerosolization properties, and bioavailability in inhalable formulations. Future studies focusing on membrane interactions, functional assays, and *in vivo* evaluations will be essential to further optimize these formulations and advance their potential as peptide-based treatments for pulmonary diseases.

## Ethical approval

This article does not contain any studies with human participants nor animals performed by any of the authors.

## Conflicts of interest

Dr Encinas-Basurto, Dr Muralidharan, Dr Islam, Dr Vallorz, Professor Black, and Professor Mansour declare no competing interests. Dr Ledford and Dr Kraft are co-founders of RaeSedo Inc., a startup company out of the University of Arizona aimed at the development of SP-A peptide-based therapeutics for the treatment of asthma.

## Data Availability

Data available upon request.
